# Considering Clinician Competencies for the Implementation of Artificial Intelligence–Based Tools in Health Care: Findings From a Scoping Review

**DOI:** 10.2196/37478

**Published:** 2022-11-16

**Authors:** Kim V Garvey, Kelly Jean Thomas Craig, Regina Russell, Laurie L Novak, Don Moore, Bonnie M Miller

**Affiliations:** 1 Center for Advanced Mobile Healthcare Learning Vanderbilt University Medical Center Nashville, TN United States; 2 Department of Anesthesiology School of Medicine Vanderbilt University Nashville, TN United States; 3 Center for Artificial Intelligence, Research, and Evaluation IBM Watson Health Cambridge, MA United States; 4 Clinical Evidence Development Aetna Medical Affairs CVS Health Hartford, CT United States; 5 Department of Medical Education and Administration School of Medicine Vanderbilt University Nashville, TN United States; 6 Department of Biomedical Informatics School of Medicine Vanderbilt University Nashville, TN United States; 7 Center of Excellence in Applied Artificial Intelligence Vanderbilt University Medical Center Nashville, TN United States

**Keywords:** artificial intelligence, competency, clinical education, patient, digital health, digital tool, clinical tool, health technology, health care, educational framework, decision-making, clinical decision, health information, physician

## Abstract

**Background:**

The use of artificial intelligence (AI)–based tools in the care of individual patients and patient populations is rapidly expanding.

**Objective:**

The aim of this paper is to systematically identify research on provider competencies needed for the use of AI in clinical settings.

**Methods:**

A scoping review was conducted to identify articles published between January 1, 2009, and May 1, 2020, from MEDLINE, CINAHL, and the Cochrane Library databases, using search queries for terms related to health care professionals (eg, medical, nursing, and pharmacy) and their professional development in all phases of clinical education, AI-based tools in all settings of clinical practice, and professional education domains of competencies and performance. Limits were provided for English language, studies on humans with abstracts, and settings in the United States.

**Results:**

The searches identified 3476 records, of which 4 met the inclusion criteria. These studies described the use of AI in clinical practice and measured at least one aspect of clinician competence. While many studies measured the performance of the AI-based tool, only 4 measured clinician performance in terms of the knowledge, skills, or attitudes needed to understand and effectively use the new tools being tested. These 4 articles primarily focused on the ability of AI to enhance patient care and clinical decision-making by improving information flow and display, specifically for physicians.

**Conclusions:**

While many research studies were identified that investigate the potential effectiveness of using AI technologies in health care, very few address specific competencies that are needed by clinicians to use them effectively. This highlights a critical gap.

## Introduction

Artificial intelligence (AI), defined as the “branch of computer science that attempts to understand and build intelligent entities, often instantiated as software programs,” [1] has been applied in the health care setting for decades. Starting in the 1960s, a cadre of computer scientists and physicians developed an interest group around AI in Medicine (AIM) [2]. By the time funding sources became aligned with opportunities in the 1980s, AI was in its “expert system” era, using rules and knowledge derived from human experts to solve problems, primarily related to medical diagnosis [3]. Projects that developed these knowledge-based systems resulted in the creation of valuable information infrastructures, including standards, vocabularies, and taxonomies that continue to anchor electronic health records (EHR) [4]. Rule-based clinical decision support (eg, case-specific clinical alerts) is an important component of today’s EHR, but it is no longer considered to be true AI [5].

Since these early forays into AI, great progress has been made in the structure and scope of information and computing technologies, as well as in data and computational resources, enabling the development of a much more powerful generation of AI tools. Human-machine collaborations exploiting these tools are already evident across professional health care practice. The ubiquitous use of personal computers and smartphones linked to external databases and highly connected AI-driven networks supports individual, team, and health system performance. This powerful new generation of AI-based tools will have wide-ranging impacts on the entire health care ecosystem, but concerns about potentially serious technical and ethical liabilities have also emerged [6].

Despite inevitable challenges, all those engaged in the practice and administration of health care should prepare for a future shaped by the presence of increasingly intelligent technologies, including robotic devices, clinical decision support systems based on machine-learning algorithms, and the flow of data and information from multiple sources, ranging from health information technology systems to individual patient sensors. While the health care and health professions education community are perched on the forefront of these complex developments, like many organizations, they may not be prepared to recognize and adequately respond to the deep-change indicators of next-generation technologies [7]. Eaneff and others recently called for new administrative infrastructures to help manage and audit the deluge of AI-induced change [8]. It is imperative for educators to be a part of that infrastructure—to actively engage in deliberations about intended changes in the working-learning environment—so that implications for learning and the needs of learners will be considered as a part of any change management process.

This impending onslaught also creates an urgent mandate for health care organizations, educators, and professional groups to consider the range of professional competencies needed for the effective, ethical, and compassionate use of AI in health care work. While numerous authors have called for structured and intentional learning programs, to date, there has been no published framework to guide teaching, learning, and assessing health care students and practitioners in this emerging and transformative domain [7,9-12]. Additionally, while there are many accredited programs (including board certification) in clinical informatics, they are focused on developing, implementing, and managing AI-based tools. However, these programs do not provide competencies for noninformatics users of AI-based tools, which represents a large gap in knowledge.

To inform these critical needs, this study aimed to systematically identify research studies that reported on provider competencies and performance measures related to the use of AI in clinical settings.

## Methods

### Study Design

A scoping review was conducted in accordance with PRISMA-ScR (Preferred Reporting Items for Systematic Reviews and Meta-Analyses extension for Scoping Reviews) [13,14] with an *a priori* protocol. The objective was to systematically identify studies that specify competencies and measure performance related to the use of AI by health care professionals. Studies had to include students or postgraduate trainees in clinical education settings across medicine, nursing, pharmacy, and social work, or practicing clinicians participating in professional development activities.

### Search Strategy

A systematic search query of MEDLINE via PubMed, CINAHL, and the Cochrane Library was conducted to identify references published or available online between January 1, 2009, and July 22, 2020 (Tables S1 to S3 in Multimedia Appendix 1). Queries including medical subject headings (MeSH) and keywords were designed around the following PICOST (population, intervention, control, outcomes, study design, and time frame) framework: (1) populations under consideration included all participants in any phase of clinical education including faculty and health care worker professional development (eg, clinical education participants in medical, nursing, or pharmacy; medical faculty and professional development; health care, clinical, or medical social workers); (2) interventions focused on AI-based tools (eg, AI terms, precision medicine, decision-making, speech recognition, documentation, computer simulation, software, patient participation or engagement, patient monitoring, health information exchange, EHR, and cloud computing) used in all settings; (3) no comparisons were required; (4) outcomes included the identification of clinical competencies and their respective measurements or domains; (5) study settings and limits included those with an abstract, conducted in humans, designed as primary studies or systematic reviews (with the same inclusion criteria), took place in US settings, and were published in English language; and (6) time—the introduction of the Health Information Technology for Economic and Clinical Health Act of 2009 was a distinguishing time point for this protocol [15,16]. AI-related tool use increased dramatically because of the organizational changes needed to accommodate meaningful use of health information technology in clinical care, justifying 2009 as a logical start point for this review.

Notably, during the protocol generation and scoping of the literature, it was determined that the MeSH term “informatics” lowered the precision (ie, irrelevant records returned) of our search strategy and greatly expanded the scope of literature to be reviewed. As such, exploded terms (eg, retrieving results under that selected subject heading and all of the more specific terms listed below in the tree) under the MeSH term “medical informatics,” including “health information exchange,” and fully exploded terms under “medical informatics applications” were applied. MeSH terms including “decision-making,” “computer-assisted,” “decision support techniques,” “computer simulation,” “clinical information systems,” and “information systems,” were among the relevant terms used. Similarly, due to imprecision, “information technology” MeSH term and “digital health” keyword were substituted with specific relevant examples for this study. Please see the search strategies provided in Tables S1 to S3 in Multimedia Appendix 1, which were created to support this scoping review protocol.

### Screening Process

Screening of each title and abstract and each full text was performed by a single reviewer for relevance against the inclusion/exclusion criteria (Table S4 in Multimedia Appendix 1).

Studies with a population exclusively limited to other types of clinicians, including allied health professionals (eg, dental hygienists, diagnostic medical sonographers, dietitians, medical assistant, medical technologists, occupational therapists, physical therapists, radiographers, respiratory therapists, and speech language pathologists), dentists, and counselors were excluded.

Relevant AI-based tools could be used in all settings (eg, outpatient, inpatient, ambulatory care, critical care, and long-term care) of clinical practice, and there was a focus on subsets that incorporated either machine learning, natural language processing, deep learning, or neural networking. Exclusions were made for AI-based tools that did not meet inclusion criteria, such as studies using technology that did not incorporate relevant AI-based tools, when the methods provided regarding the tool did not explicitly define what type of AI methodology is incorporated, or if the AI is not machine learning, natural language processing, deep learning, or neural networking. Studies on robotics (eg, robotic surgery) were excluded unless AI was a noted part of the technology.

To identify studies that specified competencies and measured performance related to the use of AI by health care professionals, the inclusion criteria (Table S4 in Multimedia Appendix 1) were limited to the 6 professional education domains of competence (eg, patient care, medical knowledge or knowledge for practice, professionalism, interpersonal and communication skills, practice-based learning and improvement, and systems-based practice) or Entrustable Professional Activities and performance. Studies were excluded if they did not report on competency-based clinical education to provide either an evaluation of a program and its outcomes related to learner achievement; a framework for assessing competency including a performance level (ie, appraisal) for each competency; or information related to instructional design, skills validation, or attitudes related to competency mastery.

The results were tracked in DistillerSR [17]. Additionally, a validated AI-based prioritization tool embedded in DistillerSR was used to support the single screening of titles and abstracts to modify or stop the screening approach once a true recall at 95% was achieved [18]. Studies had to specify competencies and measure performance related to the use of AI by health care professionals.

### Data Extraction

Data were abstracted into standardized forms (Table S5 in Multimedia Appendix 1) for synthesis and thematic analysis by 1 reviewer, and the content was examined for quality and completeness by a second reviewer, assuring that each included manuscript was dually reviewed. Abstraction for clinical education outcomes focused on how the necessary clinician competencies were described and measured. Conflict resolution was provided by consensus agreement.

### Study Quality

Study quality was assessed by dual review using the Oxford levels of evidence [19].

## Results

### Search Outcomes

**Figure 1:**
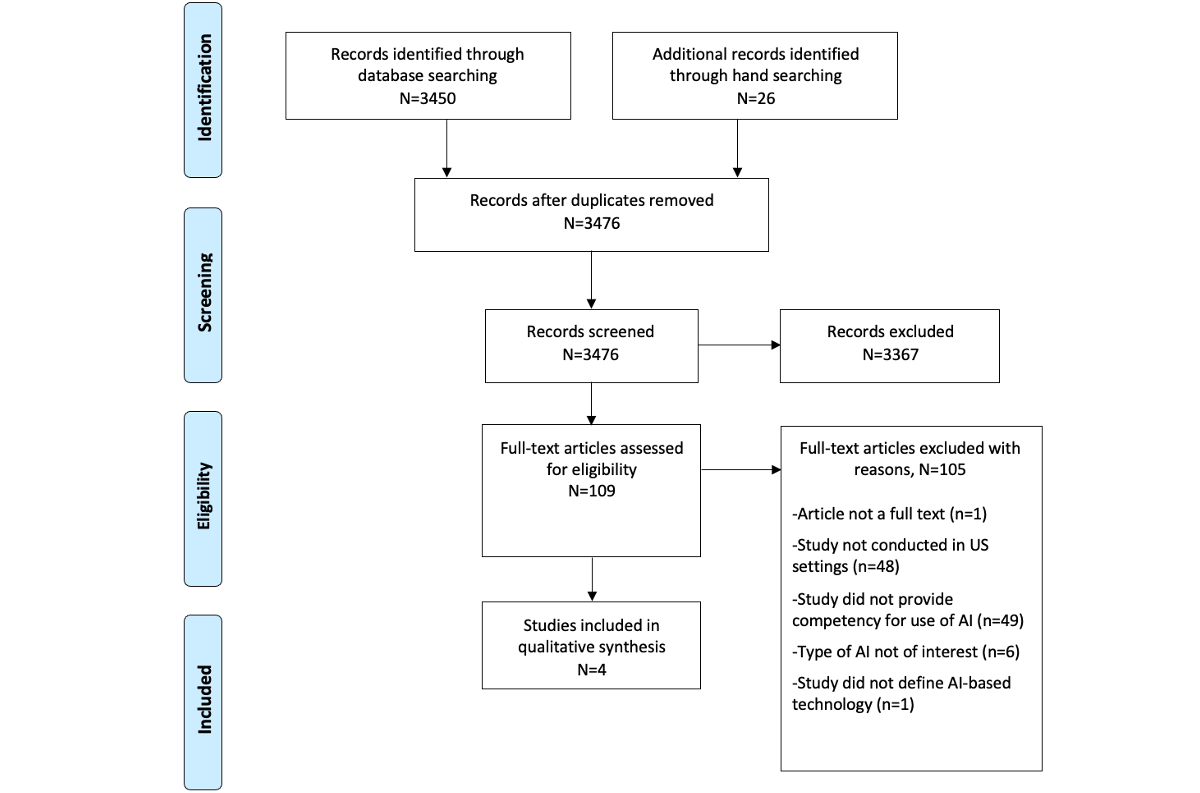
Results of literature search, the PRISMA (Preferred Reporting Items for Systematic Reviews and Meta-Analyses) diagram [14]. Summary of articles identified by systematic search queries and tracking of articles that were included and excluded across the study screening phases with reasons for exclusion of full texts provided. AI: artificial intelligence.

Literature searches yielded 3476 unique citations (Figure 1), of which 109 (3.14%) articles were eligible for full-text screening. Upon full-text screening, 4 articles met our inclusion criteria [20-23]. Abstractions of the included studies can be found in Tables 1 and 2 and Table S5 in Multimedia Appendix 1.

**Table 1:** Summary of study characteristics: design and population.

Ref. No.	Ref., Year	Design; level of evidence^a^	Clinical setting	Users of AI^b^	Stage of clinical education	Stage of clinical use	Total, n (% male)	Age (years), race or ethnicity (%)	Study duration or follow-up
1	Bien, 2018 [23]	Modeling and evaluation; *2b*^c^	Large academic hospital; imaging department	Orthopedic surgeons; general radiologists	Practicing physicians	Implementation	N/R^d^ (N/R)	N/R (N/R)	N/R
2	Hirsch, 2015 [22]	Evaluation; *4*^e^	Large private hospital; large academic medical center; nephrology and internal medicine departments	Internal medicine physicians; nephrologists	Graduate medical education (internal medicine residents and interns; nephrology fellows)	Implementation	12 (N/R)	N/R (N/R)	~9 months
3	Jordan, 2010 [21]	Evaluation; *4*	Large academic hospital; cardiothoracic intensive care department	Intensive care unit nurses	Practicing nurses	Implementation	N/R (N/R)	N/R (N/R)	N/R
4	Sayres, 2019 [20]	Experimental 3-arm observational study; *2b*	Large academic hospitals, large health systems, and specialist office; ophthalmology department	Ophthalmologists	Practicing physicians	Implementation	10 (N/R)	N/R (N/R)	N/R

^a^Adapted from Oxford Levels of Evidence [19].

^b^AI: artificial intelligence.

^d^*Level 2b*: individual cohort, modeling, or observational studies.

^c^N/R: not reported.

^e^*Level 4*: case series or poor-quality cohort studies.

**Table 2:** Summary of study characteristics: clinical competency and performance assessment.

Ref. No.	Ref., Year	Professional education domains of competence	Description (implied or explicit) of competency	User-AI^a^ interface training and description	Performance assessment
1	Bien, 2018 [23]	Patient care—clinical skills	Implied in methods; improve image interpretation	Training N/R^b^; interface not described	Metric N/P^c^; evaluate if AI assistance improves expert performance in reading MRI^d^ images
2	Hirsch, 2015 [22]	Patient care—clinical skills	Implied in methods; improve summarization of longitudinal patient record and information processing in preparation for new patients	Training N/R; authenticated user queries the database for a patient and is provided with a visual summary of content containing all visit, note, and problem information	Questionnaire; evaluate time and efficiency in information processing for patient care
3	Jordan, 2010 [21]	Communication Patient care—clinical skills Systems-based practice	Implied in methods; improve handovers in peri-operative patient care by reducing communication and informational errors	Training N/R; patient summarization and visualization tool are used as an overlay to the existing electronic patient record	Questionnaire; evaluate if AI-based tool performs better than physicians to provide clinical information and patient status in ICU^e^ handovers
4	Sayres, 2019 [20]	Patient care—clinical skills	Implied in methods; improve reader sensitivity and increase specificity of fundal images	Readers were provided training and similar instructions for use; interface not described	Metric N/P; evaluate if AI assistance increases severity grades in model predictions by assessing sensitivity and specificity of reader

^a^AI: artificial intelligence.

^b^N/R: not reported.

^c^N/P: not provided.

^d^MRI: magnetic resonance imaging.

^e^ICU: intensive care unit.

### Study Characteristics

Of the 4 studies, 3 (75%) studies were published in the past 5 years, and all 4 of the included studies were conducted in large, academic hospitals [20,22,23]. All AI-based tools in these identified studies were in a mature implementation phase and were being evaluated with either practicing physicians, residents, fellows, or nurses [20-23]. All 4 studies were undertaken to characterize the performance of internally developed niche AI software systems when used by health care professionals in specific practice settings (Table 1) [20-23].

All AI-based tools examined in these identified studies aimed to enhance an existing process, create new efficiencies, improve an outcome, and ultimately reduce cost of care [20-23]. Two of the AI-based tools were built on natural language processing frameworks [21,22] and 2 were based on deep learning processes [20,23]. One of the studies provided decision support in interpreting magnetic resonance imaging exams of the knee [23], 1 on enhancing clinician performance in detecting diabetic retinopathy [20], 1 on expediting EHR review prior to patient encounters [22], and 1 on enhancing the quality of patient handovers in the intensive care unit [21]. These systems were evaluated with measures of user satisfaction, usability, and performance outcomes. Studies used either observational or minimally controlled cohort designs, in which performance of the human-AI dyad was compared to expert performance or generalist performance alone. Three studies indicated moderate success with the AI interventions [20,21,23], and 1 had a neutral result (Table S2 in Multimedia Appendix 1) [22].

The impact of advanced data visualization, computerized image interpretation, and personalized just-in-time patient transitions are described in all 4 studies [20-23]. Competencies observed for use of these AI systems fell within the Accreditation Council for Graduate Medical Education patient care and communication competency domains [24]. However, the specific competencies clinicians required to use these innovations most effectively were not clearly described. Only 1 of the studies mentioned any form of training [20]; 3 did not describe any skill development processes for learners. None of the studies specified any need for understanding of basic AI forms, and none described the background information clinicians received about the development, training, and validation of the tools (Table 2).

### Study Quality

Using Oxford Levels of Evidence [19] to examine study quality to measure the extent that methodological safeguards (ie, internal study validity) against bias were implemented, 2 studies provided Level 2b evidence as modeling summarizations [20,23], and 2 studies provided Level 4 evidence [21,22]. The overall quality identified is moderate to low, as half of the curated evidence was classified as Level 4.

## Discussion

### Principal Findings

The volume of studies initially identified for our review confirms predictions about the growth of AI in health care. However, of these nearly 3500 articles, only 4 met the inclusion criteria. This result begs a few questions. Were our requirements overly rigorous or are the research gaps truly that numerous? Moreover, does this result reinforce concerns about a lack of organizational preparedness?

Failure to address user competencies was the most common reason for study exclusion. Many of the excluded studies compared AI tool performance with that of practicing clinicians (*human versus machine*), while others used simulations to demonstrate the potential of AI innovations to improve clinical outcomes. Only 4 research studies were identified in our search [20-23] that addressed professional competencies observed by this new AI landscape; however, none of the identified studies described new AI-related clinical competencies that had to be developed. The limited evidence derived from this review points to a large gap in adequately designed studies that identify competencies for the use of AI-based tools.

While many skills will be specific for the AI intervention being employed, these “questions of competence” are broader than the technical skills needed for use of any one AI tool or type of intelligent support [25]. All health professionals will interact with these types of technologies during their daily practice and should “know what they need to know” before using a new system. System characteristics will profoundly impact patient and clinician satisfaction as well as clinical recommendations, treatment courses, and outcomes, so health system leaders must also *know what to know* before adopting new technologies across entire health care delivery enterprises. Health care professionals at all levels have the educational imperative to articulate, measure, and iterate competencies for thriving in this evolving interface of smart technology and clinical care.

The implementation of AI into clinical workflows without sufficient education and training processes to apply the technology safely, ethically, and effectively in practice could potentially negatively impact clinical and societal outcomes. Real-world deployment of AI has caused harms due to data bias (eg, algorithms trained using biased or poor-quality data) and societal bias (eg, algorithmic output reflects societal biases of human developer) [6,26]. These biases can inflate prediction performance, confuse data interpretation, and exacerbate existing social inequities (eg, racial, gender, and socioeconomic status). These ethical considerations bring additional responsibilities and oversight of both AI-based tool implementation and its associated data to the clinical care team. The scalability of AI-based tools can also increase the scale of associated risks [8,10]. These difficulties and potential risks should be identified and understood proactively, and skills for clinicians to approach them must be included in any comprehensive training program.

The scarcity of competencies identified by this scoping review reiterates the need to develop and recommended professional competencies for the use of AI-based tools [27,28]. Ideally, these competencies should promote the effective deployment of AI in shared decision-making models that sustain or even enhance compassion, humanity, and trust in clinicians and clinical care [29]. Additionally, user-centered design (eg, more specifically, human-centered design to develop human-centric AI algorithms) should also be considered in the development of educational frameworks to support AI-related competencies required for all clinicians to use these tools effectively in clinical settings. In follow-up to this report, the authors carried out structured interviews with thought leaders to develop such a competency framework, which subsequently can be tested and iteratively refined within both simulated and authentic workplace experiences [30].

### Strengths and Limitations

This scoping review has several strengths. First, this is a novel and rigorous synthesis that adhered to PRISMA (Preferred Reporting Items for Systematic Reviews and Meta-Analyses) standards. Second, its search strategy was comprehensive and inclusive, using keywords and MeSH terms for trainee populations, settings, interventions, and outcomes that would uncover all potential accounts of currently available evidence. Moreover, the availability of these comprehensive searches will support other studies examining AI and clinical education. Third, this study included the multiple types of health care professionals who might receive training and education for the use of AI in the clinical environment.

Our results should be interpreted in the context of a few limitations. The inclusion of US-only sites limits generalizability to other global settings and health system structures. It also may have eliminated additional salient investigations, although we imagine that the dearth of US studies predicts a similar deficit from other countries. Further, due to the heterogeneity of identified interventions, it would not have been possible to compare one training approach to another. A quality assessment tool was intentionally employed, as we only planned to measure the extent that methodological safeguards (ie, internal validity) against bias were implemented. Alternatively, a risk of bias assessment would have offered a bias judgement (ie, estimation of intervention effects) on such a quality assessment, and judgement of the evidence may have shifted with this approach [31]. The search cutoff date is another limitation, as other evidence may have been published since May 2020. Other limitations include single screening of titles and abstracts, English language restriction, and exclusion of studies reported in gray literature, including conference abstracts. In addition, we excluded articles that investigated the development of robotics-assisted competencies and those that measured the impact of computer vision tools in supporting technical learning in real and simulated settings. Finally, we restricted studies to those that evaluated the use of clinical AI and excluded those supporting other learning processes, although we recognize that tools such as AI-augmented learning management systems will also become a growing part of the health professions education landscape.

### Conclusions

While many research studies were identified that investigate the potential effectiveness of using AI technologies in health care, very few address specific competencies that are needed by clinicians to use them effectively. This highlights a critical gap.

## Appendix

Multimedia Appendix 1 Supplementary tables.

## Abbreviations

AI: artificial intelligence
EHR: electronic health records
MeSH: medical subject headings
PICOST: population, intervention, control, outcomes, study design, and time frame
PRISMA: Preferred Reporting Items for Systematic Reviews and Meta-Analyses
PRISMA-ScR: Preferred Reporting Items for Systematic Reviews and Meta-Analyses extension for Scoping Reviews
