# Supplementary material for: Considering Clinician Competencies for the Implementation of Artificial Intelligence–Based Tools in Health Care: Findings From a Scoping Review
Source: JMIR Med Inform. 2022 Nov 16;10(11):e37478. doi: 10.2196/37478 (PMC9713618; doi:10.2196/37478)
Supplement: Multimedia Appendix 1 [file medinform_v10i11e37478_app1.docx]

**Multimedia Appendix 1**

Table S1. MEDLINE search (via PubMed)

| **Search no.** | **Facet** | **Search Terms** | **PUBMED Search Results (April 28, 2020)** |
| --- | --- | --- | --- |
| **POPULATION: Healthcare professionals and clinical education** | | | |
| 1 | Medical education participants | medical education OR  medical student* OR (graduate AND med*) OR intern* OR resident* OR fellow* OR “Education, Medical”[MeSH] OR  physician* OR continuing medical education | 2,977,549 |
| 2 | Medical faculty and professional development | (medical AND faculty AND professional development) OR “Faculty, Medical”[MeSH] OR “Education, Professional, Retraining”[MeSH] | 17,571 |
| 3 | Nursing education participants | nursing education OR “Nursing Education”[MeSH] OR continuing nursing education OR “Nurses”[MeSH] OR “Licensed practical nurses”[MeSH]  OR nurse* OR nurse practitioner* OR nurse anesthetist* OR nurse clinician* OR nurse midwife OR nurse midwive* OR licensed practical nurse* OR registered nurse* | 505,121 |
| 4 | Pharmacy education participants | pharmacy education OR continuing pharmacy education OR “Pharmacy Education”[MeSH] OR “Pharmacists”[MeSH]  or pharmacist* | 63,229 |
| 5 | Healthcare/clinical/medical social worker | (“Social Workers”[MeSH] AND (clinical or medical or healthcare or health care)) OR (Healthcare AND social worker*) OR (clinical AND social worker*) OR (medical AND social worker*) | 6,482 |
| 6 | All participants in the phases of clinical education including faculty and healthcare worker professional development | #1 OR #2 OR #3 OR #4 OR #5 | 3,409,632 |
| **INTERVENTIONS: Artificial intelligence (AI)-based tools used in all settings** | | | |
| 7 | AI terms | artificial intelligence OR “AI” OR machine learning OR “ML” OR deep learning OR learning OR machine intelligence OR computational intelligence OR algorithm* OR model* OR “Artificial Intelligence”[MeSH] OR  heuristic* OR expert system* OR knowledge base* OR (biological AND ontology) OR natural language processing OR “NLP” OR neural network* OR automation OR transactional system* OR (knowledge acquisition AND computer) OR (knowledge representation and computer) | 166,782 |
| 8 | Precision medicine | precision medicine OR personalized medicine OR individualized medicine OR P health OR P-Health OR personalized genomics OR “Precision Medicine”[MeSH] | 84,051 |
| 9 | Decision-making | computer aided detection OR (computer assisted AND decision) OR (computer-assisted AND decision) OR decision making OR decision-making OR (imaging AND deep learning) OR medical imag* OR  computer vision OR computer vision system* OR computer reasoning OR “Decision Making, Computer-Assisted”[MeSH] OR “Molecular Imaging”[MeSH] OR  (decision AND (support OR aid* OR analys?s OR model* OR technique OR rule* OR prediction)) OR predictive analytic* OR “Decision Support Systems, Clinical”[MeSH] OR  “Decision Support Techniques”[MeSH] | 721,801 |
| 10 | Speech recognition | speech recognition OR voice recognition OR (software AND (voice OR speech) AND recognition) OR user-computer interface OR ambient intelligence* OR ambient-assisted OR (conversational AND (artificial intelligence or “AI”)) OR chatbot* OR conversational agent* OR dialogue system* OR “Speech Recognition Software”[MeSH] OR  “User-Computer Interface”[MeSH] | 52,471 |
| 11 | Documentation | documentation OR (“Documentation”[MeSH] AND artificial intelligence OR natural language processing OR deep learning OR machine learning OR neural network) | 1,084,101 |
| 12 | Computer simulation | computer simulation OR augmented reality OR virtual reality OR physiome* OR (patient AND model*) OR biological model* OR in silico* OR computerized model* OR mixed reality OR mixed realities OR augmented realities OR virtual reality OR “Computer Simulation”[MeSH] | 987,912 |
| 13 | Software | application programming interface* OR  (software AND (machine learning OR neural network* OR natural language processing or artificial intelligence OR deep learning)) | 17,083 |
| 14 | Patient -participation / engagement | (patient AND (engagement OR involvement OR empowerment OR participation OR activation OR compliance OR non-adherence OR non-compliance OR cooperation OR mobile technology OR telemedicine OR telehealth OR mobile health OR mHealth OR eHealth OR m-Health OR mobile-health OR telecommunication OR “Mobile Applications”[MeSH] OR “Telemedicine” [MeSH] OR “Patient portals”[MeSH]) AND (machine learning OR neural network* OR natural language processing or artificial intelligence OR deep learning)) OR  ((app OR application*) n3 (smartphone* OR smart-phone OR mobile* OR phone*)) OR patient web portal OR patient web-portal OR patient portal OR web portal OR “Patient Participation”[MeSH] OR “Patient Compliance”[MeSH] | 119,400 |
| 15 | Patient Monitoring | ((machine learning OR neural network* OR natural language processing or artificial intelligence OR deep learning) AND (quantified self OR connected health OR big data OR gamification OR social media OR health 2.0 OR fitness tracker* OR activity tracker* OR wearable* OR patient monitor* OR activity monitor* OR sensor* OR physiologic monitoring OR internet of things OR “IOT” OR “Fitness Trackers”[MeSH] OR “Social Media”[MeSH] OR “Monitoring, Physiologic”[MeSH] OR “Internet of Things”[MeSH])) | 16,184 |
| 16 | Health information exchange | health information exchange OR electronic health information OR electronic health communication OR interoperability OR “HIE” OR “Health information exchange”[MeSH] OR “Health information interoperability”[MeSH] | 83,357 |
| 17 | Electronic health records | electronic health record* OR personal health record* OR electronic medical record* OR “EMR” OR “EHR” OR “Electronic health records”[MeSH] OR “Health records, personal”[MeSH] | 25,536 |
| 18 | Cloud computing | cloud computing OR cloud process OR cognitive computing OR “Cloud computing”[MeSH] | 5,144 |
| 19 | Combine all AI domains | #7 OR #8 OR #9 OR #10 OR #11 OR #12 OR #13 OR #14 OR #15 OR #16 OR #17 OR #18 | 2,909,984 |
| 20 | Use AI terms to include content | #19 AND (machine learning OR neural network* OR natural language processing or artificial intelligence OR deep learning) | 150,422 |
| 21 | Combine clinical education and AI-based tools | #6 AND #20 | 18,355 |
| **OUTCOMES: Competencies and Performance** | | | |
| 22 | Competence, Clinical competency | competenc* OR competency-based OR sub-competenc* OR learning outcome* OR learning objective OR clinical skills OR “Professional Competence”[MeSH] OR Competency-Based Education"[MeSH] | 278,928 |
| 23 | Measurement / Domains | evaluation OR program evaluation OR educational evaluation OR assessment OR needs assessment OR self-assessment OR performance OR improvement OR “Academic Performance”[MeSH] OR “Needs Assessment”[MeSH] OR “Ethics, Professional”[MeSH] OR “Computer User Training”[MeSH] | 4,100,909 |
| 24 | Combine clinical education outcomes | #22 OR #23 | 4,262,907 |
| 25 | Identify Competency-Based Clinical Education Outcomes Regarding AI-Based Tools | #21 AND #24 | 8,003 |
| 26 | Identify narrative reviews | Review[pt] NOT (Cochrane OR systematic or meta-analy*) | 2,348,428 |
| 27 | Exclude narrative reviews | #25 NOT #26 | 7,557 |
| 28 | Identify case studies, editorials, and letters | "case reports"[pt] OR "letter"[pt] OR "editorial"[pt] OR "case series" | 3,523,639 |
| 29 | Exclude case studies, editorials, and letters | #27 NOT #28 | 7,457 |
| 30 | Exclude *in vitro* and *in vivo* studies | #29 NOT ("in vitro" OR "in vivo") | 7,317 |
| 31 | Limit to United States | #30 AND (North America[MeSH:noexp] OR United States[MeSH]) OR (#29 NOT (Africa[MeSH] OR Asia[MeSH] OR Australia[MeSH] OR Canada[MeSH] OR Europe[MeSH] OR South America[MeSH])) | 6,691 |
| 32 | Limit to articles with abstract | #31 Filters: Abstract available | 6,642 |
| 33 | Limit to articles published since 2009 | #31: Abstract available, published between January 1, 2009 and April 28, 2020 | 5,171 |
| 34 | Limit to English | #31 Filters: Abstract available, published in English | 5,128 |
| 35 | Limit to studies in humans | #31 Filters: Abstract available, published in English, and studies in humans | 2,999 |

Table S2. CINAHL search

| **Search no.** | **Facet** | **Search Terms** | **CINAHL Search Results (April 28, 2020)** |
| --- | --- | --- | --- |
| **POPULATION: Healthcare professionals and clinical education** | | | |
| 1 | Medical education participants | (MM "Education, Continuing") OR (MM "Continuing Education Providers") OR (MM "Education, Medical, Continuing") OR (MM "Education, Nursing, Continuing") OR (MM "Education, Health Sciences") OR (MM "Education, Medical") | 18,066 |
| 2 | Medical faculty and professional development | (MH "Curriculum+") OR (MH "Education, Clinical+") OR (MM "Education, Competency-Based") OR (MH "Faculty") OR (MM "Academic Performance") OR (MM "Computerized Educational Testing") OR (MM "Credentialing Examinations") OR (MM "Professional Competence") OR (MH "Educational Technology") OR (MM "Faculty, Medical") OR (MM "Faculty, Nursing") OR (MM "Faculty Development") OR (MH "Knowledge") OR (MM "Professional Knowledge") OR (MH "Learning Methods+") OR (MH "Program Development+") OR (MH "Staff Development") OR (MH "Teaching") OR (MM "Computer User Training") OR (MM "Models, Educational") OR (MM "Teaching Methods, Clinical") | 147,803 |
| 3 | Nursing education participants | (MM "Education, Nursing, Practical") OR (MM "Education, Nurse Anesthesia") OR (MM "Education, Nursing, Continuing") OR (MM "Education, Nursing, Doctoral") OR (MM "Education, Nursing, Masters") OR (MH "Advanced Practice Nurses+") OR (MH "Case Managers") OR (MM "Associate Degree Nurses") OR (MM "Baccalaureate Nurses") OR (MM "Doctorally Prepared Nurses") OR (MM "Diploma Nurses") OR (MM "Masters-Prepared Nurses") OR (MM "Nurse Researchers") OR (MH "Nurses by Specialty+") OR (MM "Faculty, Nursing") | 44,338 |
| 4 | Pharmacy education participants | (MM "Education, Pharmacy") OR (MM "Continuing Education Providers") | 819 |
| 5 | Healthcare/clinical/medical social worker | ((MH "Health Personnel") OR (MM "Case Managers") OR (MM "Community Health Workers") OR (MM "Social Workers")) AND (healthcare OR medical OR clinical")) | 15,744 |
| 6 | All participants in the phases of clinical education including faculty and healthcare worker professional development | #1 OR #2 OR #3 OR #4 OR #5 | 203,457 |
| **INTERVENTIONS: Artificial intelligence (AI)-based tools used in all settings** | | | |
| 7 | AI terms | (MH "Artificial Intelligence+") OR (MM "Expert Systems") OR (MM "Knowbots") OR (MH "Knowledge Bases+") OR (MH "Machine Learning+") OR (MM "Natural Language Processing") OR (MM "Neural Networks (Computer)" | 9,069 |
| 8 | Precision medicine | (MM "Individualized Medicine") OR  precision medicine OR personalized medicine OR individualized medicine OR P health OR P-Health OR personalized genomics OR "Precision Medicine"[MeSH] | 1,852 |
| 9 | Decision-making | (MH "Decision Making, Computer Assisted+") OR (MM "Decision Support Systems, Clinical") OR (MH "Decision Support Techniques+") OR (MH "Surgery, Computer-Assisted+") OR (MH "Image Processing, Computer Assisted") OR (MH "Decision Making, Organizational") OR (MH "Image Interpretation, Computer Assisted") OR (MH "Radiographic Image Interpretation, Computer-Assisted") | 17,699 |
| 10 | Speech recognition | (MH "User-Computer Interface+") OR (MM "Voice Recognition Systems") OR (MM "Speech Intelligibility") | 7,575 |
| 11 | Documentation | (MM "Charting") OR (MM "Coding") OR (MM "Medical Records+") OR (MM "Medical Transcription") OR (MM "Medical Orders") OR (MM "Nursing Orders") OR (MH "Policy and Procedure Manuals") OR (MH "Electronic Health Records+") OR (MM "Medical Record Linkage") OR (MM "Medical Records, Personal") OR (MM "Nursing Records") OR (MM "Patient Discharge Summaries") OR (MM "Problem Oriented Records") OR (MM "Record Review") | 31,225 |
| 12 | Computer simulation | MH "Computer Simulation+") OR (MM "Augmented Reality") OR (MH "Virtual Reality+") OR (MM "Virtual Reality Exposure Therapy") | 8,014 |
| 13 | Software | (MH "Software+") AND (machine learning OR neural network* OR natural language processing or artificial intelligence OR deep learning))" OR "application programming interface* | 1 |
| 14 | Patient -participation / engagement | ((MM "Patient Portals") OR (MM "Mobile Applications") OR (MM "Telemedicine") OR (MM "Telenursing") OR (MM "Text Messaging") OR (MM "Interactive Voice Response Systems") OR (MM "Email") OR (MM "Instant Messaging") OR (MM "Internet") OR (MM "Remote Consultation") OR (MM "Telepathology") OR (MM "Teleradiology") OR (MM "Telerehabilitation") OR (MM "Telepsychiatry") AND (machine learning OR neural network* OR natural language processing or artificial intelligence OR deep learning)) | 26,984 |
| 15 | Patient Monitoring | ((MM "Monitoring, Physiologic") OR (MH "Social Media+") OR (MH "Biophysical Instruments+") OR (MH "Wearable Sensors+") OR (MM "Accelerometers") OR (MM "Blood Glucose Meters") AND (machine learning OR neural network* OR natural language processing or artificial intelligence OR deep learning)) | 24,049 |
| 16 | Health information exchange | (MM "Health Information Networks") OR (MH "Clinical Information Systems+") | 26,678 |
| 17 | Electronic health records | MM "Electronic Health Records” | 8,252 |
| 18 | Cloud computing | MH "Cloud Computing+" | 1,103 |
| 19 | Combine all AI domains | #7 OR #8 OR #9 OR #10 OR #11 OR #12 OR #13 OR #14 OR #15 OR #16 OR #17 OR #18 | 126,080 |
| 20 | Use AI terms to include content | #19 AND (machine learning OR neural network* OR natural language processing or artificial intelligence OR deep learning) | 4,569 |
| 21 | Combine clinical education and AI-based tools | #6 AND #20 | 242 |
| **OUTCOMES: Competencies and Performance** | | | |
| 22 | Competence, Clinical competency | (MM "Education, Competency-Based") OR (MH "Teaching Methods+") OR (MM "Outcomes of Education") OR (MM "Clinical Competence") | 86,685 |
| 23 | Measurement / Domains | (MM "Needs Assessment") OR (MM "Student Performance Appraisal+") OR (MM "Competency Assessment") OR (MH "Teaching+") OR (MH "Program Development") OR (MH "Learning Methods+") | 185,433 |
| 24 | Combine clinical education outcomes | #22 OR #23 | 193,860 |
| 25 | Identify Competency-Based Clinical Education Outcomes Regarding AI-Based Tools | #20 AND #24 | 138 |
| 26 | Limit to United States | #25 with Limiters: Geographic subset, USA | 43 |
| 27 | Limit to articles with abstract and exclude PubMed articles | #25 with Limiters: Abstract available and exclude articles in PubMed | 38 |
| 28 | Limit to articles published since 2009 | #25 with Limiters: Abstract available, published between January 2009 and May 2020 | 36 |
| 29 | Limit to English | #25 with Limiters: Abstract available, published in English | 36 |
| 30 | Limit to studies in humans | #25 with Limiters: Abstract available, published in English, and studies in humans | 20 |

Table S3. Cochrane Library search

| **Search no.** | **Facet** | **Search Terms** | **COCHRANE Library Search Results (April 28, 2020)** |
| --- | --- | --- | --- |
| **POPULATION: Healthcare professionals and clinical education** | | | |
| 1 | Medical education participants | [mh "Education, Medical, Continuing"] OR [mh "Education, Medical, Graduate"] OR medical education OR  medical student* OR (graduate AND med*) OR intern* OR resident* OR fellow* OR physician* | 269,499 |
| 2 | Medical faculty and professional development | (medical AND faculty AND professional development) OR [mh "Faculty, Medical] OR [mh “Education, Professional, Retraining”] | 468 |
| 3 | Nursing education participants | nursing education OR [mh “Nursing Education”] OR continuing nursing education OR [mh “Nurses”] OR [mh “Licensed practical nurses”]  OR nurse* OR nurse practitioner* OR nurse anesthetist* OR nurse clinician* OR nurse midwife OR nurse midwive* OR licensed practical nurse* OR registered nurse* | 31,189 |
| 4 | Pharmacy education participants | pharmacy education OR continuing pharmacy education OR [mh “Pharmacy Education”] OR [mh “Pharmacists”]  or pharmacist* | 5,887 |
| 5 | Healthcare/clinical/medical social worker | ([mh “Social Workers”] AND (clinical or medical or healthcare or health care)) OR (Healthcare AND social worker*) OR (clinical AND social worker*) OR (medical AND social worker*) | 2,555 |
| 6 | All participants in the phases of clinical education including faculty and healthcare worker professional development | #1 OR #2 OR #3 OR #4 OR #5 | 290,663 |
| **INTERVENTIONS: Artificial intelligence (AI)-based tools used in all settings** | | | |
| 7 | AI terms | artificial intelligence OR “AI” OR machine learning OR “ML” OR deep learning OR learning OR machine intelligence OR computational intelligence OR algorithm* OR model* OR [mh “Artificial Intelligence"] OR  heuristic* OR expert system* OR knowledge base* OR (biological AND ontology) OR natural language processing OR “NLP” OR neural network* OR automation OR transactional system* OR (knowledge acquisition AND computer) OR (knowledge representation and computer) | 241,437 |
| 8 | Precision medicine | precision medicine OR personalized medicine OR individualized medicine OR P health OR P-Health OR personalized genomics OR [mh "Precision Medicine”] | 131,703 |
| 9 | Decision-making | computer aided detection OR (computer assisted AND decision) OR (computer-assisted AND decision) OR decision making OR decision-making OR (imaging AND deep learning) OR medical imag* OR  computer vision OR computer vision system* OR computer reasoning OR [mh "Decision Making, Computer-Assisted"] OR [mh "Molecular Imaging"] OR  (decision AND (support OR aid* OR analys?s OR model* OR technique OR rule* OR prediction)) OR predictive analytic* OR [mh “Decision Support Systems, Clinical"] OR  [mh "Decision Support Techniques"] | 48,592 |
| 10 | Speech recognition | speech recognition OR voice recognition OR (software AND (voice OR speech) AND recognition) OR user-computer interface OR ambient intelligence* OR ambient-assisted OR (conversational AND (artificial intelligence or “AI”)) OR chatbot* OR conversational agent* OR dialogue system* OR [mh "Speech Recognition Software"] OR  [mh "User-Computer Interface"] | 2,488 |
| 11 | Documentation | documentation OR [mh "Documentation"] AND (artificial intelligence OR natural language processing OR deep learning OR machine learning OR neural network) | 87 |
| 12 | Computer simulation | computer simulation OR augmented reality OR virtual reality OR physiome* OR (patient AND model*) OR biological model* OR in silico* OR computerized model* OR mixed reality OR mixed realities OR augmented realities OR virtual reality OR [mh "Computer Simulation"] | 58,648 |
| 13 | Software | application programming interface* OR  (software AND (machine learning OR neural network* OR natural language processing or artificial intelligence OR deep learning)) | 565 |
| 14 | Patient -participation / engagement | (([mh "Mobile Applications"] OR [mh "Telemedicine"] OR [mh "Patient portals"] OR [mh "Patient Participation"] OR [mh "Patient Compliance"] OR engagement OR involvement OR empowerment OR participation OR activation OR compliance OR non-adherence OR non-compliance OR cooperation OR mobile technology OR telemedicine OR telehealth OR mobile health OR mHealth OR eHealth OR m-Health OR mobile-health OR telecommunication) AND patient AND (machine learning OR neural network* OR natural language processing or artificial intelligence OR deep learning)) | 478 |
| 15 | Patient Monitoring | ((machine learning OR neural network* OR natural language processing or artificial intelligence OR deep learning) AND (quantified self OR connected health OR big data OR gamification OR social media OR health 2.0 OR fitness tracker* OR activity tracker* OR wearable* OR patient monitor* OR activity monitor* OR sensor* OR physiologic monitoring OR internet of things OR “IOT” OR [mh "Fitness Trackers"] OR [mh "Social Media"] OR [mh "Monitoring, Physiologic"] OR [mh "Internet of Things"]) | 985 |
| 16 | Health information exchange | health information exchange OR electronic health information OR electronic health communication OR interoperability OR “HIE” OR [mh “Health information exchange"] OR [mh “Health information interoperability"] | 16,547 |
| 17 | Electronic health records | electronic health record* OR personal health record* OR electronic medical record*OR “EMR” OR “EHR” OR [mh “Electronic health records"] OR [mh “Health records, personal"] | 12,254 |
| 18 | Cloud computing | cloud computing OR cloud process OR cognitive computing OR [mh “Cloud Computing"] | 209 |
| 19 | Combine all AI domains | #7 OR #8 OR #9 OR #10 OR #11 OR #12 OR #13 OR #14 OR #15 OR #16 OR #17 OR #18 | 380,706 |
| 20 | Use AI terms to include content | #19 AND (machine learning OR neural network* OR natural language processing or artificial intelligence OR deep learning) | 3,848 |
| 21 | Combine clinical education and AI-based tools | #6 AND #20 | 1,313 |
| **OUTCOMES: Competencies and Performance** | | | |
| 22 | Competence, Clinical competency | competenc* OR competency-based OR sub-competenc* OR learning outcome* OR learning objective OR clinical skills OR [mh “Professional Competence”] OR [mh “Competency-Based Education"] | 33,034 |
| 23 | Measurement / Domains | evaluation OR program evaluation OR educational evaluation OR assessment OR needs assessment OR self-assessment OR performance OR improvement OR [mh “Academic Performance”] OR [mh “Needs Assessment”] OR [mh “Ethics, Professional”] OR [mh “Computer User Training”] | 492,325 |
| 24 | Combine clinical education outcomes | #22 OR #23 | 504,286 |
| 25 | Identify Competency-Based Clinical Education Outcomes Regarding AI-Based Tools | #21 AND #24 | 1,096 |
| 26 | Limit to United States | #25 AND ([mh ^"North America"] OR [mh "United States"]) OR  (#25 NOT ([mh "Africa"] OR [mh "Asia"] OR [mh "Australia"] OR [mh "Canada"] OR [mh "Europe"] OR [mh "South America"])) | 1,080 |
| 27 | Limit to articles with abstract | #26 Filters: Cochrane Reviews | 465 |
| 28 | Limit to articles published since 2009 | #30: Abstract available, published between January 1, 2009 and April 28, 2020 | 434 |

**Table S4. Inclusion/Exclusion Criteria using PICOST Framework**

| **PICOST** | **Inclusion** | **Exclusion** |
| --- | --- | --- |
| Population | Healthcare professionals will include physicians, physician assistants, pharmacists, registered nurses, advanced practice nurses (e.g., nurse midwives, nurse anesthetists, CNAs), healthcare social workers.  All participants in the phases of clinical education including:   - degree programs - post-graduate training - continuing professional development / continuing education - faculty professional development | Other types of clinicians including allied health professionals (e.g., dental hygienists, diagnostic medical sonographers, dietitians, medical assistant, medical technologists, occupational therapists, physical therapists, radiographers, respiratory therapists, speech language pathologists), dentists, and counselors. |
| Intervention | Artificial intelligence (AI)-based tools used in all settings (e.g., outpatient, inpatient, ambulatory care, critical care, long-term care) of clinical practice with a focus on these subsets that incorporate either machine learning, natural language processing, deep learning, or neural networking:   - Adaptive Learning - Clinical Documentation - Cloud Computing - Computer Simulation - Decision-making - Electronic Health Records - Health information exchange - Patient monitoring - Patient-participation / engagement - Precision Medicine - Software - Speech Recognition | Studies using technology that do not incorporate AI-based tools and/or the methods provided regarding the tool do not explicitly define what type of AI methodology is incorporated or if the AI is not machine learning, natural language processing, deep learning, or neural networking.  Studies on robotics (e.g., robotic surgery) will be excluded unless noted that AI is part of technology. |
| Comparison | No comparisons are required. | N/A |
| Outcomes | Study describes professional education domains of competence that include:   - Patient Care - Clinical skills - Clinical reasoning - Inquiry skills - Medical Knowledge / Knowledge for Practice - Professionalism - Interpersonal and Communication Skills - Practice-Based Learning and Improvement - Systems-Based Practice   The study reports on domains of competencies listed above or Entrustrable Professional Activities (EPAs) AND performance. | The study does not report on competency-based clinical education to provide either an evaluation of a program and its outcomes related to learner achievement; a framework for assessing competency including a performance level (i.e., appraisal) for each competency; or information related to instructional design, skills validation, or attitudes related to competency mastery. |
| Situation | Studies conducted in the United States. | The study setting is conducted outside of the United States. |
| Limits – study type | The publication describes a primary study or systematic review (with the same inclusion criteria) with or without meta-analyses. All study designs will be included. | The publication describes a study design other than a primary study or systematic review. Case studies, case series, narrative reviews, editorials, or policy pieces.  Conference abstracts will be excluded. |
| Limits - language | The publication is in English. | The publication is in a language other than English. |
| Limits - human | The study is conducted in humans. | The study is not conducted in humans or examines human *in vitro* cells. |
| Limits – time; published in the last 11 years | The study was published in the last 11 years (January 1, 2009 and May 1, 2020) | The publication date is before January 1, 2009 or after May 1, 2020. |

**Table S5.** Summary of study characteristics: artificial intelligence and clinical impact(s)

| **Ref.**  **No.** | **Ref., Year** | **Study Objective** | **Type(s) of AI** | **Brand;**  **Name of AI** | **Description of**  **AI-based Tool** | **Anticipated Clinical Impact** | **Observed Clinical**  **Utility of AI-based Tool** |
| --- | --- | --- | --- | --- | --- | --- | --- |
| 1 | Bien, 2018 [23] | Test the performance of an AI-based model for identifying knee MRI pathologies against radiologists interpretations; test performance (accuracy, specificity, and sensitivity) of clinicians when using model in practice. | Deep learning model (DLM) including convolutional neural network (CNN) | N/R; MRNet | To develop and assess the predictive model and clinical experts, 1,370 knee MRI exams were prospectively labeled from clinical reports. DICOM images were abstracted, preprocessed and linked to reports. The algorithm was prepared using the following data sets: trained (1,130 exams and 1,088 patients) tuning set (120 exams, 111 patients), internal validation (120 exams, 113 patients), external validation (using exams in a 60:20:20 ratio into training, tuning, and validation sets with stratified random sampling. The validation exams were annotated by musculoskeletal radiologists, model-unassisted clinical experts, and model-assisted clinical experts. | Use of MRNet as an interpretative tool along with clinical expert revie–­w, would reduce the number of patients referred for knee surgery and reduce the time spent completing MRI image interpretation. | 1) Model provided increase in clinical experts’ (both surgeons and radiologists) mean specificity (4.8%) in identifying knee pathologies (*P*<0.001), which equates to 3 less patients having unnecessary surgery;  2) model provided increase in general radiologists specificity in identifying knee pathologies (*P*=0.003);  3) model assistance improved inter-rater reliability of identifying knee pathologies (*P* N/R). |
| 2 | Hirsch, 2015 [22] | Evaluate the effectiveness of a point-of-care patient summarization and visualization tool that uses AI components. | Natural language processing (NLP); problem salience computation | HARVEST; UMLS (SNOMED-CT); distributed infrastructure - Apache Hadoop, Apache Hbase | HARVEST architecture has two online processing modules to function on top of any clinical system: HL7 message and visit parsing from EHR and web-based visualization for each patient. The back-end processing allows for parsing of visit information and indexing of clinical notes, and salience computation for cloud visualization of problems. Salience weights were based on the TF*IDF framework. Clinical notes were processed using NLP by UMLS/SNOWMED-CT concepts and semantic groupings. Apache Hadoop was used to scale parsing and salience computation, and processed notes, extracted problems, salience weights, and meta-data were stored on Apache Hbase. The front-end visualization used HTML5 canvas and Javascript code. | Provides cognitive support for physicians (and potentially other providers) in reviewing patient histories, preparing for visits, and follow-ups. | There was no difference in the accuracy of summaries (*P*=0.85) or time to use (*P*=0.959) the existing medical record system with HARVEST.  No correlation of HARVEST with user satisfaction (r=0.379, *P*=0.224). |
| 3 | Jordan, 2010 [21] | Evaluate the use of an advanced clinical decision support tool that is used to summarize individual patient’s clinical abnormalities and illness severity. | Natural language generation, knowledge-based graphics generation, knowledge representation and reasoning systems; intelligent agents, medical logic modules, and text generators (“inference engine”) | Multimedia Abstract Generation of Intensive Care (MAGIC) | MAGIC consists of several distributed system components that are integrated by Inter-Language Unification package that work on subtasks to generate a multimedia briefing. MAGIC is composed of intelligent agents, medical logical modules, and text generators (collectively called the “inference engine”) for planning and generating briefings in text, speech, and graphics. | MAGIC provides context-specific information for ICU nurses to better prepare for incoming patients:  1) a summary for a cardiac patient's operative course in multimedia format meant to guide continuum of care;  2) a point-in-time “inference engine" depiction of a patient's clinical status. | MAGIC provided 200% more information at the patient’s arrival compared to physician’s bedside briefings; lines, blood products, and anesthetics informational categories were significant (all *P*<0.01) differences in the distribution of data.  The inference engine was tested as a quality assurance tool to identify abnormal events; accuracy of abnormal event identification was increased by 11% (*P* N/R) compared to physician assessment.  Quality of transmitted data regarding the content of the bedside briefing filtered transcripts:  1) recall by physicians (60%) was limited compared to MAGIC (78%);  2) precision by physicians (45%) was limited compared to MAGIC (100%).  MAGIC system had 94% approval with 96% accuracy rating by admitting nurses. |
| 4 | Sayres, 2019 [20] | Use AI-based models to predict diabetic retinopathy (DR) severity for the improvement of grading performance by a physician reader. | Models: 1) Deep-learning algorithm with and without heatmap explanation; 2) Clinician plus deep-learning algorithm with and without heatmap explanation.  Machine learning model | N/R; TensorFlow deep learning algorithm | 1) Algorithm trained using Inception version 4 model architecture and dataset with 1.6 million retinal fundus images;  2) Tuning set - image set of 2,000 total 45° fundus images with reference standards generated by 3-fellowship trained retina specialists; heatmap generated by trained algorithm with tuning set; 3) Evaluation set filtered from trained and tuned dataset; 4) Application of the algorithm to explain severity level as a score based on strength of evidence for each DR class. | 1) Augment non-specialist clinicians reading of images for better referral decisions at scale; 2) Improve non-physician and non-specialist reader image interpretation;  3) Improve consistency of care and prevent attentional lapses; | Model had 5-class (fraction of reads in which the 5-class International Clinical DR grade exactly match reference standard) accuracy of 88.4% (95% CI, 87.9-88.9%); 96.9% (95% CI, 96.3-97.3%) accuracy for images with no DR; and 57.9% (95% CI, 55.3%-60.6%) accuracy for images with mild or worse NPDR.  Algorithmic assistance increased sensitivity (reader, 79.4% vs. algorithm 91.5%) for DR without reducing specificity (reader, 96.6% vs. algorithm, 94.7%).  Both types of model assistance improved reader performance accuracy, but increase was associated with the background of the reader; retina specialists performed as well as the algorithm (*P*=0.06), but general ophthalmologists were less accurate than the algorithm (*P*<0.001).  Model increased self-reported confidence (all *P*<0.05).  Model experience and use decreased grading time (*P*=0.006); decrease in grading time was stronger in assisted vs. unassisted conditions (*P*<0.001). |

Footnotes:

Abbreviations and symbols – AI, artificial intelligence; CI, confidence interval; CNN, convolutional neural network; DLM, deep learning model; DR, diabetic retinopathy; ICU, intensive care unit; MAGIC; multimedia abstract generation of intensive care; MRI, magnetic resonance imaging; MRNet, MRI convolutional neural network; NLP, natural language processing; NPDR, mild nonproliferative diabetic retinopathy; N/R, not reported; SNOWMED-CT, systematicized nomenclature of medicine – clinical terms; *P*, P value; TF*IDF, term frequency inverse to document frequency; UMLS, unified medical language system; vs, versus; °, degree
